# Human resource information systems in health care: a systematic evidence review

**DOI:** 10.1093/jamia/ocw141

**Published:** 2016-10-05

**Authors:** Aizhan Tursunbayeva, Raluca Bunduchi, Massimo Franco, Claudia Pagliari

**Affiliations:** 1 Department of Economics, Management, Society and Institutions, University of Molise, Campobasso, Italy; 2 Business School, University of Edinburgh, Edinburgh, UK,; 3 eHealth Research Group, Usher Institute of Population Health Sciences and Informatics, University of Edinburgh, Edinburgh, UK

**Keywords:** eHealth, health care management, information systems, systematic review, human resource information systems

## Abstract

**Objective:** This systematic review aimed to: (1) determine the prevalence and scope of existing research on human resource information systems (HRIS) in health organizations; (2) analyze, classify, and synthesize evidence on the processes and impacts of HRIS development, implementation, and adoption; and (3) generate recommendations for HRIS research, practice, and policy, with reference to the needs of different stakeholders.

**Methods:** A structured search strategy was used to interrogate 10 electronic databases indexing research from the health, social, management, technology, and interdisciplinary sciences, alongside gray literature sources and reference lists of qualifying studies. There were no restrictions on language or publication year. Two reviewers screened publications, extracted data, and coded findings according to the innovation stages covered in the studies. The Critical Appraisal Skills Program checklist was adopted to assess study quality. The process of study selection was charted using a Preferred Items for Systematic Reviews and Meta-Analysis (PRISMA) diagram.

**Results:** Of the 6824 publications identified by the search strategy, 68, covering 42 studies, were included for final analysis. Research on HRIS in health was interdisciplinary, often atheoretical, conducted primarily in the hospital sector of high-income economies, and largely focused uncritically on use and realized benefits.

**Discussion and Conclusions:** While studies of HRIS in health exist, the overall lack of evaluative research raises unanswered questions about their capacity to improve quality and efficiency and enable learning health systems, as well as how sociotechnical complexity influences implementation and effectiveness. We offer this analysis to decision makers and managers considering or currently implementing an HRIS, and make recommendations for further research.

**Trial Registration:** International Prospective Register of Systematic Reviews (PROSPERO): CRD42015023581. http://www.crd.york.ac.uk/PROSPERO/display_record.asp?ID=CRD42015023581#.VYu1BPlVjDU.

## INTRODUCTION

### Administrative information systems as a topic of research in health

Administrative information systems (IS) in health organizations deal with such processes as records management, billing and finance, and aspects of human resource management (HRM), which can also help to support care delivery, quality improvement, and research. Despite their role as enablers of efficient, effective, and, potentially, “learning” health organizations,^1^ administrative systems have been somewhat neglected as a topic of research in health informatics.^2^ This systematic review focuses on a key subcategory of administrative systems, human resource information systems (HRIS).

### What HRIS are and why they are so important

Staff costs account for 65–80% of health organizations’ total operating budgets.^3^ Therefore, effective management of human resources (HR) is essential, from both a clinical and financial perspective. HRIS support a variety of HRM practices, including recruitment and performance management, and provide health leaders with crucial information guiding effective capacity planning and resource allocation. HRIS can take various forms, ranging from dedicated stand-alone packages (eg, payroll) to components of integrated enterprise resource planning (ERP) or hospital information systems (HISs). Not perceived as life-critical, HRIS have received very little attention in the health informatics literature, and their development, implementation, use, and impacts in health organizations are poorly understood compared with clinical systems (eg, electronic health records). HRIS research also tends to be distributed across the social (encompassing business and management), information and communications technology (ICT), and health sciences literature.

### Why a systematic evidence review of HRIS in health care is needed

Although forms of HRIS have been used in the health sector for almost half a century,^4^ this is still an evolving area. Increasingly sophisticated modular HRIS are being procured and implemented in health organizations worldwide,^5^ often at high expense in terms of technology, support, and change management. While the benefits of these systems have been much vaunted by HRIS vendors^6^ and policy makers,^7^ there have also been spectacular failures, where large-scale implementations have encountered huge overspends, weak organizational buy-in, or poor interoperability with existing systems.^8^ Given the opportunity costs of getting these projects wrong, developers, procurers, and managers require more guidance on the usefulness, effectiveness, and implementation barriers associated with HRIS, as well as how to evaluate them. Thus this systematic review is very timely.

### What is new about this review

Our scoping study identified only 2 previous literature reviews specifically examining HRIS in health, both of which were limited in scope.^9^ We therefore conducted an interdisciplinary systematic review utilizing sources of evidence from the ICT, social science, and health research literature, encompassing any ICT used for HR administration, management, and development practices in health organizations. The specific objectives were to: (1) determine the prevalence and scope of existing research and evaluation pertaining to HRIS in health organizations; (2) analyze, classify, and synthesize existing evidence on the processes and impacts of HRIS development, implementation, and use; and (3) generate recommendations for HRIS research, practice, and policy, with reference to the needs of different stakeholders and communities of practice.

## METHODS

### Search strategy

A comprehensive search strategy was developed and tested iteratively during a scoping phase (see Supplementary Appendix 1). This was used to interrogate 10 international online databases indexing medical/health (Cochrane Library, MEDLINE, EMBASE); social science (ABI/INFORM, ASSIA, Sociological Abstracts), ICT (IEEE Xplore); and multidisciplinary research (Scopus, Web of Science Core Collection, ScienceDirect). Gray literature sources were also examined, including reports from the World Health Organization (WHO), relevant professional organizations (eg, Chartered Institute of Personnel and Development, Society for Human Resource Management, Healthcare Information and Management Systems Society), and consulting firms (eg, Deloitte, Ernst & Young, PricewaterhouseCoopers, KPMG). Academic dissertations were searched via Google, and the reference lists of qualifying articles were searched by hand to identify additional relevant studies. No restrictions were applied to publication year or language.

### Article screening and selection

#### *Procedure*

Outputs were stored in EPPI-Reviewer 4 software. After initial screening of titles and abstracts, the full text of potentially relevant articles was examined by 2 reviewers (AT, RB) to assess their fit with the inclusion criteria. Disagreements were resolved through consensus or arbitration by a third reviewer (CP).

#### *Inclusion criteria*

There were 2 inclusion criteria: (1) studies involving a formal or semiformal approach to the investigation or evaluation of HRIS, whether led by academia or industry (eg, consulting sector), or from within the health sector; and (2) studies of broader business/administrative/ERP/HIS systems that explicitly examine their application to HR practices.

#### *Exclusion criteria*

We excluded descriptive reports, pure market research, articles focused on software design issues, studies that were not primarily focused on HRIS or that mentioned HRIS without specifying the health sector, and articles examining generic ERP/HIS without referring to HR functionalities. Details of the filters applied at each screening stage are included in the PRISMA flow diagram.

### Data extraction and analysis

One author (AT) extracted information from all eligible studies using a structured form containing the following fields: authors, publication year, setting (type of organization, country/region in which the study was conducted), innovation stage, journal discipline, HRIS functionality, research purpose/questions, theoretical basis, HRIS users, study design, and main findings. Extracted information was then verified by all team members (CP, RB, and MF).

To differentiate among HRIS project stages, we borrowed from existing innovation models (eg^10^^,^^11^) and coded the results according to 3 main innovation stages: (1) development (eg, needs assessment, procurement initiation, prototyping, and user acceptance testing), (2) implementation (eg, purchasing, systems integration, organizational change management, and training), and (3) use (including adaptation of organizational procedures to accommodate routinization of the innovation as part of day-to-day working practices).

We also coded studies using Parry and Tyson’s^12^ framework to compare the intended and actual benefits of HRIS adoption. This includes 6 types of goals relating to operational efficiency, service delivery, strategic orientation, manager empowerment, standardization, and organizational image. Additional goals emerging from our analysis were added into separate categories.

Finally, of the various models of HRM practices described in the literature (eg^13^), including in relation to HRIS (eg^5^), we chose to adapt Foster’s E-HRM Landscape model^14^ to classify our studies (see Figure 3), as it covers the majority of the HRM practices mentioned in the reviewed articles. To the verbs describing core objectives of HRIS in the e-HRM Landscape we added “interact,” taking account of HRIS modules described as self-service, HR portals, or HR Intranets. We also added several subcategories reflecting additional functions mentioned in the studies (eg, employee relations and qualifications tracking).

### Critical appraisal techniques

Following recommendations for systematic reviews of qualitative research,^15^^,^^16^ we adapted the qualitative Critical Appraisal Skills Programme checklist.^17^ Questions concerning the appropriateness of qualitative methodology and ethical issues were eliminated, since a first reading of the material revealed that most eligible studies were qualitative and lacked ethical considerations (see Supplementary Appendix 2). In addition to the “yes” or “no” answers, we added a “not clear” option (corresponding to scores of 1.0, 0.5, and 0, respectively). One reviewer (AT) appraised all eligible studies. A second reviewer (CP) independently appraised a random 20% sample to assess interrater consistency and facilitate discussion about the process and any ambiguities. Since only a few minor discrepancies were identified, a secondary appraisal focused on studies about which the first reviewer was uncertain.

## RESULTS

In all, 6824 results were generated by the search strategy and 6104 titles and abstracts remained after removing 720 duplicates. Of these, 399 qualified for full-text review, 232 due to their potential eligibility and 167 because there was insufficient information in the title or abstract to make a decision. After removing documents that did not meet the inclusion criteria, 68 publications representing 42 separate studies were included in the final analysis (see Table 1

**Table 1.** Characteristics of the included studies

#	Authors, year (discipline)	Country (income^a^); HO (IS)	Research goals	Study design	Quality score (0–10)	Innovation stage	Outcomes reported
S1	Altuwaijri and Khorsheed, 2012^18^ (social science)	Saudi Arabia (high); Mixed^b^ (gen.: ERP)	To propose a new generic model for successful implementation of IT projects	Qual.	4	Implementation	Barriers: individual, and project
						Use	Realized benefits^c^: operational, strategic, empowerment, and IT infrastructure
S2	Bakar, Sheikh and Sultan, 2012^19^ (ICT/health)	Tanzania (low); Ministry of Health (ded.: open-source HRIS)	To describe the opportunities and related challenges of integrating an open-source software process in the organization	Qual.	5.5	Use	Barriers: environment, project, and individual
							Realized benefits^c^: operational, and service
							Approaches to: technology
S3	Bondarouk and Ruel, 2003^20^ (N/A)	Netherlands (high); secondary (hospital) (ded.: personnel and salary administration system)	To explore differences in the adoption of a human management system between 2 groups of users	Qual.	6	Implementation	Facilitators: individual, technology, and organization
	Bondarouk and Sikkel, 2003^21^ (N/A)		To apply a theory of a group learning to highlight relevant aspects of implementation of groupware				Barriers: organization, and individual
	Bondarouk and Sikkel, 2004^22^ (N/A)		To look closer at groupware implementation from a learning-oriented approach				
	Bondarouk, 2004^23^(social science/ICT)		To describe a project concerning the implementation of a personnel management system			Use	Facilitators: individual, technology, and organization
	Bondarouk and Sikkel, 2005^24^ (social science)		To validate 5 processes of adoption of IT through group learning, and to get insights on which of the group processes are most influential in the system implementation				
	Bondarouk and Ruel, 2008^25^ (N/A)		To explore the relationship between the organizational climate for innovation and ICT implementation success				
	Bondarouk and Ruel, 2008^26^ (social science)		To describe an HRM system that can lead to IT implementation success				Barriers: organization, and individual
S4	IntraHealth Int., Inc.,^d^ 2009^27^ (N/A)	Nine African countries (low or lower-middle); NHS (ded.: open-source HRIS)	To present an overview of the results achieved by the Capacity project	Report (Qual.)	5.5	Use	Facilitators: project
							Realized benefits^c^: strategic, and interest from other countries
S5	Cockerill and O’Brien-Pallas, 1990^28^ (health)	Canada (high); secondary (>1 hospitals) (gen.: nursing workload measurement systems)	To develop a profile of use of nursing workload measurement systems in Canadian hospitals, assess user satisfaction, and identify challenges/perceived problems and research issues related to these systems	Quant.	6	Implementation	Barriers: organization
							Generic: project, and individual
	O’Brien-Pallas and Cockerill, 1990^29^ (health)		To explore senior nurse executives’ needs and expectations for nursing workload systems			Use	Realized benefits^c^: strategic
							Satisfaction: familiarity with the system, its functions or use of them, and user satisfaction varied between roles; system needs to reflect true workload for users to be satisfied
							Approaches to: technology, and individual
S6	Dent et al., 1991^30^ (N/A)	UK (high); secondary (>1 hospitals) (ded.: manpower IS)	To find out how district managements had prepared for and were responding to implementation of 3 corporate computer systems	Qual.	5.5	Implementation	Facilitators: organization, and project
							Barriers: organization
	Dent, 1991^31^ (social science)						Approaches to: project, and technology
			To examine the development of computing and IT strategies within NHS England and Wales				
S7	Engbersen, 2010^32^ (N/A)	Netherlands (high); secondary (hospital) (gen.: Intranet)	To advance understanding of the special features of e-HRM implementation and provide insight into the influences e-HRM has on the HRM department and the organization with its HR activities	Qual.	6.5	Implementation	Recommendations: individual, organization, task, and project
						Use	Barriers: individual, project, task, and organization
							Outcomes > generic: no change to operational, and strategic
S8	Escobar-Perez and Escobar-Rodriguez, 2010^33^ (social science)	Spain (high); secondary (hospital) (gen.: ERP)	To analyze the process of implementation of ERP systems in hospitals as an organization with divided and heterogeneous functional areas, and to identify the principal technological objectives that were set in the process of implementation, which of those objectives were achieved, and the deficiencies that subsequently became evident	Qual.	5.5	Development	Expected benefits^c^: strategic Generic: organization, technology, and individual
						Implementation	Generic: individual
							Approaches to: individual, inter-organization, and project
	Escobar-Perez et al., 2010^34^ (ICT)					Use	Barriers: project, and individual
							Satisfaction: varies between roles
							Approaches to: technology
S9	Evers, 2009^35^ (N/A)	Netherlands (high); secondary (hospital) (ded.: HR portal)	To assess the contribution of an HR portal toward HR processes	Qual.	6.5	Development	Expected benefits^c^: strategic, service, and operational
						Implementation	Recommendations: project, task, and individual
						Use	Realized benefits^c^: empowerment
							Satisfaction: users need time to judge system; strong relationship between system ease of use and user satisfaction
							Outcomes > generic: no change to operational, and service
							Downsides: reduced operational, and empowerment
							Recommendations: project, and task
S10	Fahey and Burbridge, 2008^36^ (health)	USA (high); secondary (>1 hospitals) (gen.: daily staff management system)	To present a case study of a failed attempt to apply the principles of diffusion of innovation to a software program	Qual.	4.5	Development	Generic: technology
						Implementation	Facilitators: organization
							Barriers: technology, and organization
						Use	Facilitators: organization
							Barriers: organization, and task
S11	Fehse, 2002^37^ (N/A)	Netherlands (high); secondary (hospital) (ded.: personnel IS)	To explore to what extent and how organizational politics explain IS implementation outcomes	Qual.	6.5	Development	Expected benefits^c^: strategic
						Implementation	Facilitators: individual
							Barriers: organization, project, and individual
							Generic: individual, and organization
							Approaches to: project, and technology
						Use	Outcomes > generic: no change to operational
S12	Gurol et al., 2010^38^ (N/A)	Turkey (upper-middle); secondary (hospital) (ded.: e-HRM)	To investigate several specific and critical points that will contribute to a better understanding of e-HRM and provide a model for implementation of e-HRM	Qual.	4.5	Use	Realized benefits^c^: operational, strategic, and empowerment
S13	Hawker et al., 1996^39^ (health)	Canada (high); secondary (hospital) (gen.: workload measurement system)	To describe the development and application of a computerized workload measurement tool for use in hospital nursing education departments	Qual.	2.5	Use	Realized benefits^c^: service, and strategic
S14	Helfert, 2009^40^ (social science)	Ireland (high); NHS (ded.: personnel payroll attendance and recruitment system)	To outline a framework for analyzing health care process management projects	Qual.	5.5	Implementation	Barriers: individual, project, task, inter-organization, organization, and technology
							Approaches to: inter-organization and project
S15	Kazmi and Naaranoja, 2014^41^ (social science)	Pakistan (lower-middle); secondary (hospital) (ded.: HRIS)	To propose an evaluation of how, in a small-business scenario, bits and pieces of knowledge can be seen scattered at different work locations and how management can strategically arrange and manage a viable data resource in the form of existing knowledge base to be retrieved as and when required	Quant.	4	Use	Satisfaction: majority of users satisfied with information system provides
S16	Kumar et al., 2013^42^ (health)	Pakistan (lower-middle); NHS (NS: HRIS)	To document how HR information is currently being collected, managed, and reported; to identify the gaps related to HRH information that need to be urgently addressed; and to suggest the tools and processes for managing HR data	Quant.	6.5	Development	Expected benefits^c^: operational, service, and strategic
S17	Lin et al., 2010^43^ (ICT/health)	Taiwan (high); secondary (hospital) (gen.: nursing assistant management system)	To compare the results of manual operation and system intervention in assigning work to nursing assistants, in order to evaluate the system’s performance	Mixed method	4.5	Use	Realized benefits^c^: operational, and patient care
							Satisfaction: different categories of users are satisfied with the system
S18	Memel et al., 2001^44^ (health)	USA (high); secondary (>1 hospitals) (gen.: Intranet)	To discuss specific components of the information management and IT infrastructure, examples of the impacts they have on patients, caregivers, and the organization, and lessons learned	Qual.	2	Development	Expected benefits^c^: operational
						Use	Realized benefits^c^: operational, and service
							Approaches to: technology
S19	Parry and Tyson, 2011^12^ (social science)	UK (high); secondary (>1 hospitals) (ded.: e-HRM)	To examine the goals stated by organizations for introduction of e-HRM, whether they were actually achieved, and the factors affecting this	Qual.	7	Development	Expected benefits^c^: operational, service, strategic, standardization, and empowerment
						Implementation	Facilitators: individual, and project
							Generic: technology
						Use	Realized benefits^c^: operational, service, strategic, and standardization
S20	Pierantoni and Vianna, 2003^45^ (health/social science)	Brazil (upper-middle); Departments of Health (NS: HRIMS)	To evaluate implementation of HRIS in selected health departments and present the implementation evaluation methodology; and to identify the limits and possibilities for using the system as an HR planning and management tool in local health systems	Mixed method	5.5	Development	Expected benefits^c^: strategic
						Implementation	Facilitators: environment, and organization
							Barriers: environment, organization, technology, and individual
						Use	Facilitators: environment and organization
							Approaches to: task
S21	PWC, 2010^46^ (N/A)	Queensland, Australia (high); NHS (ded.: payroll system)	To review the organization of corporate services under the shared services model and determine the most appropriate arrangements for the future; to investigate and make recommendations on the appropriate governance model for shared services going forward; and to provide recommendations for the future rollout of the Corporate Solutions Program and the most effective way to deliver it	Report (Qual.)	5.5	Development	Expected benefits^c^: strategic and standardization
	KPMG, 2010^47^ (N/A)		To summarize the work undertaken to date on the review of the Queensland Health (QH) payroll implementation project				Facilitators: individual, and project
	KPMG, 2010^48^ (N/A)						Recommendations: project, technology, environment, task, organization, and individual
	KPMG, 2012^49^ (N/A)		To review the current status, proposed solutions, strategies, programs of work, and governance frameworks in place for the QH payroll system				Approaches to environment
	E&Y, 2010^50^ (N/A)		To conduct a review of QH payroll and rostering systems to establish their ongoing suitability for QH, and to ascertain what potential options are available to resolve the recently experienced payroll problems			Implementation	Facilitators: project, and individual
	Auditor-General of Queensland, 2010^51^ (N/A)		To evaluate the effectiveness of the Department of Public Works’s program and project management and QH processes in relation to the business readiness of and transition to new systems				Barriers: environment, inter-organization, organization, project, technology, task, and individual
	Chesterman, 2013^52^ (N/A)		To present a full and careful inquiry into implementation of the QH payroll system				Approaches to: project, inter-organization, and technology
	Silva and Rosemman, 2012^53^ (N/A)		To propose an approach to represent the dynamic relations between social and material entities where the latter are divided into technical and organizational entities	Qual.			Recommendations: inter-organization, project, task, and technology
	Eden and Sedera, 2014^54^ (N/A)		To illustrate the factors that contributed to QH’s disastrous implementation project; and to understand the broader applications of this project failure on state and national legislations as well as industry sectors			Use	Generic: organization, project, and technology
	Thite and Sandhu, 2014^8^ (social science/ICT)		To ascertain the main reasons for the failure of the new payroll implementation project; and to develop a theoretically and practically derived system development life cycle model				Approaches to: project
							Outcomes > generic: resignation of Minister of Health, strikes, improved country ICT strategy, and governance procedures
							Recommendations: inter-organization, organization, project, task, technology, and individual
S22	Rauhala, 2008^55^ (N/A)	Finland (high); secondary mixed (gen.: patient classification system)	To evaluate whether the patient classification system was valid and feasible enough to be used as a measurement tool for HRM in nursing in the wards of somatic specialized health care	Quant.	7.5	Use	Approaches to: task
	Fagerstrom et al., 2000^56^ (health)						
	Fagerstrom et al., 2000 ^57^ (health)						
	Rauhala and Fagerstrom, 2004^58^ (health)						
	Rauhala and Fagerstrom, 2007^59^ (health)						
	Rauhala et al., 2007^60^ (health)						
S23	Fagerstrom, 2009^61^ (health)	Finland (high); secondary (>1 hospitals) (gen.: patient classification system)	To illustrate how the system can be used to facilitate evidence-based HRM	Quant.	6	Use	Realized benefits^c^: strategic
							Approaches to: task
S24	Rainio and Ohinmaa, 2005^62^ (health)	Finland (high); secondary (hospital) (gen.: patient classification system)	To assess the feasibility of the system in nursing staff management, and whether it can be seen as the transferring of nursing resources between wards according to the information received from nursing care intensity classification	Quant.	5.5	Use	Approaches to: technology
S25	Riley et al., 2007^63^ (health)	Kenya (lower-middle); NHS (ded.: nursing workforce database)	To describe the development, initial findings, and implications of a national nursing workforce database system in Kenya	Mixed method	5	Use	Facilitators: environment, and organization
							Realized benefits^c^: strategic
							Approaches to: technology
							Recommendations: technology
S26	Riley et al., 2012^64^ (health/social science)	Int.; NHS (NS: HRIS)	To review and assess national practices in HRIS implementation worldwide; identify the main areas of weakness in HRIS implementation, with attention to countries facing acute health workforce shortages; and draw upon documented best practices to offer recommendations to decision and policy makers on how to improve the science and application of HRIS	Systematic review	6.5	Development	Expected benefits^c^: strategic
						Use	Approaches to: environment, organization, technology, and task
S27	Rodger et al., 1998^65^ (N/A)	USA (high); mixed (ded.: HRIS)	To describe the efforts of the HR department to redesign its HRIS to better meet enterprise-wide goals of cost effectiveness and efficiency	Mixed method	4.5	Use	Satisfaction: users satisfied with distribution and collection of HRIS reports and their confidentiality, but not with complicated procedures and forms for HRIS
	Rodger et al., 1998^66^ (social science/ICT)						Approaches to: technology, and task
							Recommendations: project, task, and individual
S28	Ruland, 2001^67^ (ICT/health)	Norway (high); secondary (hospital) (gen.: decision support system)	To describe the system development process	Mixed method	5.5	Development	Expected benefits^c^: strategic, empowerment, and operational
							Facilitators: project, and individual
	Ruland and Ravn, 2001^68^ (ICT/health)		To evaluate the system’s effect on nursing costs, quality of management information, user satisfaction, and ease of use, and its usefulness as decision support for improved financial management and decision-making			Implementation	Facilitators: project, and individual
						Use	Facilitators: organization, individual, project, and technology
							Realized benefits^c^: operational, and strategic
							Satisfaction: users satisfied with system, and information it provides
S29	Sammon and Adam, 2010^69^ (social science/ICT)	Ireland (high); NHS (gen.: ERP)	To investigate the managers’ level of understanding of ERP project implementation and the preparations that should be made to increase the likelihood of success	Qual.	6.5	Development	Expected benefits^c^: strategic
						Implementation	Barriers: project
							Approaches to: organization, and project
S30	Schenck-Yglesias, 2004^70^ (N/A)	Malawi (low); NHS (gen.: HRIS)	To review the availability of staff deployment and training data from routine IS in Malawi and inform the Ministry of Health and Population of deficiencies that would need to be addressed to better inform the development and ongoing monitoring and deployment of training policies and plans	Report (Qual.)	5.5	Development	Approaches to: inter-organization, and technology
						Use	Recommendations: task
S31	Shukla et al.,^d^ 2014^71^ (N/A)	India (lower-middle); NHS (ded.: open-source HRIS)	To review HRIS across all 28 states and 7 union territories of India to assess their purpose, scope, coverage, software technology, usability, and sustainability	Report (Qual.)	5.5	Development	Expected benefits^c^: operational, and compliance
							Facilitators: project
						Use	Approaches to: inter-organization, project, task, and individual
S32	Smith et al., 1979^72^ (ICT)	USA (high); secondary (hospital) (ded.: computer-based scheduling system)	To discuss 3 years’ experience in computer-assisted scheduling of nursing personnel	Qual.	2.5	Development	Expected benefits^c^: strategic
						Implementation	Facilitators: individual, and project
							Approaches to: technology, and individual
						Use	Realized benefits^c^: operational, and empowerment
							Satisfaction: can decline over time due to technical design, operation and organization changes, and changed capabilities of users
							Approaches to: technology, and individual
							Recommendations: environment, organization, and project
S33	Spaulding, 2012^73^ (N/A)	USA, Australia, Canada, UK (high); secondary (>1 hospitals) (NS: HRIS)	To review existing conceptualizations of HRIS and set forth propositions defining the impact such systems have on individual and organizational performance; to test several of those propositions by evaluating hospital HRIS use and hospital-acquired condition outcomes; and to conduct cost effectiveness analysis examining the compositions of rapid response teams	Quant.	6.5	Use	Realized benefits^c^: patient care
S34	Spero et al., 2011^74^ (health/social science)	Uganda (low); professional organization (ded.: open-source HRIS)	To describe Uganda’s transition from a paper filing system to an electronic HRIS; and to describe how HRIS data can be used to address workforce planning questions via an initial analysis of the Uganda Nurses and Midwives Council training, licensure, and registration records	Mixed method	5	Use	Realized benefits^c^: operational, and patient care
							Approaches to: technology
							Recommendations: technology
S35	Stamouli and Mantas, 2001^75^ (ICT/health)	Greece (high); secondary (>1 hospitals) (gen.: IS for the nursing service)	To describe the development and evaluation of an IS for the Nursing Service Administration	Quant.	4.5	Development	Expected benefits^c^: strategic, and operational
							Barriers: individual, and organization
						Use	Facilitators: technology, and project
							Satisfaction: users satisfied with system user friendliness, and information it provides
S36	Thouin and Bardhan, 2009^76^ (N/A)	USA (high); secondary (>1 hospitals) (ded.: HRM systems)	To study the effect of IT usage on quality improvements in patient outcomes and examine the effect of clinical and administrative IT adoption and usage on financial performance	Quant.	6	Use	Realized benefits^c^: patient care, and operational
S37	Valentine et al., 2008^77^ (health)	USA (high); secondary (>1 hospitals) (ded.: automated open-shift management program)	To discuss how a successful nursing initiative to apply automation to open-shift scheduling and fulfillment across a 3-hospital system had a broad enterprise-wide impact	Mixed method	2	Implementation	Facilitators: individual
							Approaches to: task
						Use	Realized benefits^c^: operational, empowerment, and strategic
							Approaches to: technology
S38	Waring, 2000^78^ (N/A)	UK (high); secondary (hospital) (ded.: payroll-personnel system)	To critically investigate potential emancipatory principles for system analysis, design, and development synthesized from the wider literature, then translate these principles into practice within the context of IS implementation	Qual.	7	Development	Expected benefits^c^: service, compliance, and factors beyond organization’s control
							Facilitators: project
							Barriers: organization, task, and inter-organization
							Approaches to: inter-organization, and project
	Waring, 2004^79^ (social science)					Implementation	Barriers: organization, and inter-organization
							Approaches to: project, and technology
S39	Warner et al., 1991^80^ (health)	USA (high); secondary (>1 hospitals) (ded.: nurse scheduling system)	To describe what nursing administration is looking for in an automated scheduling system; and to discuss issues of implementation from the viewpoint of nursing administration, including realizable benefits	Qual.	2	Use	Realized benefits^c^: strategic, and operational
S40	Waters et al., 2013^81^ (health)	Kenya (lower-middle); NHS (ded.: open-source HRIS)	To document the impact of system data on HR policy, planning, and management	Mixed method	5.5	Use	Realized benefits^c^: operational, strategic, and compliance
S41	West et al., 2004^82^ (health)	UK (high); primary (gen.: IS to collect workload data)	To describe the implementation of a computerized IS to collect workload data and discuss feedback from staff evaluation of use and value	Qual.	5.5	Use	Barriers: organization, task, and individual
S42	WHO, 1990^83^ (N/A)	Int.; NHS(NS: HRH IS)	To share expertise and experiences in the areas of research and health personnel IS and identify strategies for better use of information and research in decision-making for HRH development	Report (Qual.)	5.5	Development	Expected benefits^c^: strategic
							Facilitators: environmental
							Approaches to: environment and inter-organization

^a^Classified according to the World Bank’s Country and Lending Groups.^84^^b^Primary and secondary. ^c^Benefits: operational = operational efficiency; service = service delivery; strategic = strategic orientation; empowerment = empowerment of managers and employees; compliance = statutory compliance.

*Abbreviations*: HO = health organization; IT = information technology; Qual. = qualitative; Quant. = quantitative; NHS = National Health System; Int. = international; HRH = Human Resources for Health; HRIMS = human resource information and management system; gen. = generic IS; ded. = dedicated IS; NS = not specified; N/A = not applicable.

**Figure 1.**
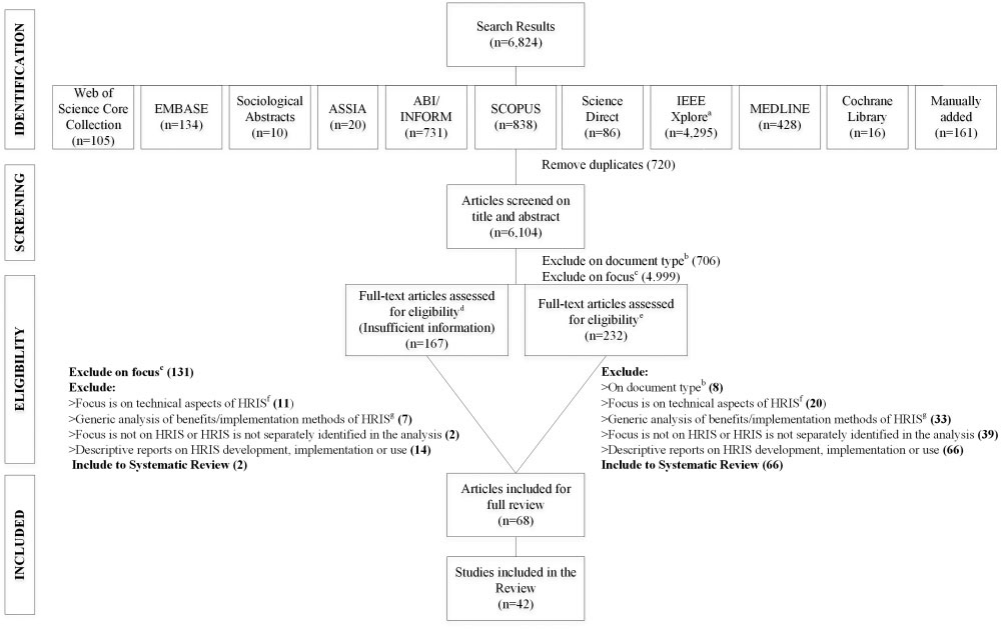
PRISMA flow diagram. ^a^Database has limitations on the number of keywords, therefore the search had to be run several times to ensure that all search query keywords were included (please see^9^). ^b^Book reviews, front and back covers, copyright notice, title pages, collection of conference proceedings’ descriptions, tables of contents, press releases, announcements, descriptions of issues, advertisements, bulletins, questionnaires, notices of retraction, chair’s messages, keynotes, plenary talks, welcome messages, news published in journals and magazines that have “news” in their title and news published by companies that do not provide any analytical or research materials, presentation description, very brief cases and analytical materials published in newspaper and magazines, company profiles, advertising/marketing articles. ^c^Articles not related to HRIS in health organizations, research on HR practices in health organizations that do not defer to use of ICT in relation to HR activities. ^d^Articles where no abstract was available or where title and abstract did not give sufficient detail to judge eligibility, articles on HRIS that do not specify the industry/sector in which they were implemented, articles on generic ERP/HIS that do not specify the module/functionality and/or industry/sector in which they were implemented. ^e^Potentially relevant articles referring to HRIS in health organizations. ^f^Articles focused on computer science models (eg, software specification) or management science models (eg, creating algorithms to enable staffing and scheduling in health organizations). ^g^Generic analyses of principles, benefits, requirements, implementation methods of HRIS in health organizations, or pure market research.

). The stages of selection are illustrated in the PRISMA diagram labeled Figure 1

.

### Publication characteristics

**Figure 2.**
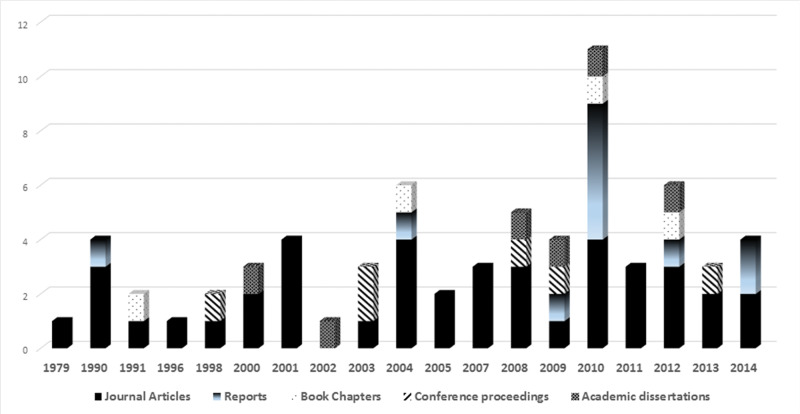
Types of publications on HRIS by year.

Included articles were published between 1979 and 2014. More than half entered the literature within the last decade, peaking in 2010, when 11 were published (see Figure 2).

**Figure 3.**
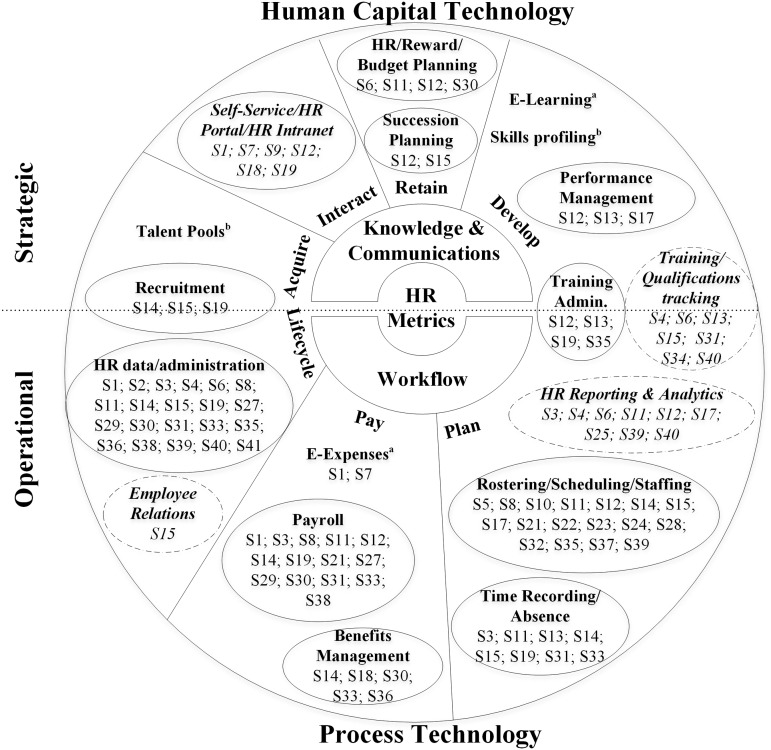
HRM practices examined in the included studies. ^a^Out of scope of this review (please see^9^). ^b^Not mentioned in any of the qualifying studies. Solid line ovals: existing Foster’s e-HRM landscape categories. Dashed line ovals, text in italic: categories added to Foster’s e-HRM landscape.

Out of 68 publications, the vast majority (n = 41) were journal articles. To test our observation that HRIS in health is a multidisciplinary topic,^9^ these articles were first classified into subject areas according to the Scimago Journal ranking portal (Scimagojr) and afterward using broader discipline categories such as health, ICT, and social science. Nine articles were classified manually, as the journals were not covered by Scimagojr. 29 articles (71%) were published in a single discipline: 18 in health (44%), 9 in social science (22%), and 2 in ICT (5%). Just under a third (29%) were published in multidisciplinary journals, including 5 covering ICT and health (12%), 3 covering health and social science (7%), and 4 covering social science and ICT (10%).

### Country

The majority of studies were conducted in high-income countries (see Table 1): 17 in Europe (4 each in the Netherlands and the UK, 3 in Finland, 2 in Ireland, and 1 each in Greece, Norway, Spain, and Turkey), 9 in North America (7 in the United States and 2 in Canada), and 1 in Australia (although several authors independently studied this case, it was classified as one study). Only 4 studies were conducted in Asia (2 in Pakistan and 1 each in India and Taiwan), 6 in Africa (2 in Kenya, 1 each in Malawi, Uganda, and Tanzania, and 1 covering 9 African countries). One study was conducted in South America (Brazil), and 1 in the Middle East (Saudi Arabia). Three studies either involved several countries across different regions or did not specify the countries covered.

### Units of analysis

Although diverse health organizations were represented, more than half of the studies focused on hospitals in high-income countries, typically taking one hospital as their unit of analysis. Only one study focused on a primary health care organization (see Table 1). Studies in low-income countries mostly reviewed country-wide HRIS and/or systems developed, implemented, and used by government Departments of Health or professional organizations.

### Research designs and study quality

Most studies (n = 24) used qualitative methods. Nine employed quantitative designs, while 8 used mixed methods. One study was a systematic literature review (a second review identified by our search did not meet the inclusion criteria; it focused on ICT for enabling continuing professional development, and e-learning was out of the scope of this review^9^).

Descriptive studies were excluded at the full-text review stage. None of the qualifying studies received a maximum score of 8 on quality assessment. Those scoring highest were quantitative studies and postgraduate research theses; those scoring lower did not adequately explain their units of analysis, research methodology, or sources of potential bias. Of the qualitative studies, very few scored higher than 6 (see Table 1 and Supplementary Appendix 2).

### Theoretical frameworks

Over half of the studies (n = 22) did not specify any theoretical perspective. The other 20 referred to a diversity of frameworks, most specifying only one (see Table 2

**Table 2.** Theoretical frameworks referred to in qualifying studies

Disciplinary perspective	Framework	Study
HR and HR related	Concept of experiential learning	S3
	Central principles of HRM	S22
	Personnel as resource in HRM theory	S23
	HRIS impact through drawing from motivation in organizational behavior and theory of work performance	S33
Innovation and change	Diffusion of innovations	S10
	Theoretical models of organizational change	S11
IS and IS related	InnoDiff model based on model for IS success	S1
	Framework of impacts of technology implementation	S8
	Technology acceptance model	S9
	Corporate information factory	S18
	System development life cycle	S21
	Concept of mindfulness to develop concept of preparedness in ERP implementation	S29
	Process-centric role of ICT in terms of its impact on business value	S36
Specific combinations of HR and IS concepts	Conceptual framework developed by WHO Study Group linking 3 components: decision-making in the development of HR for health, research, and IS	S42
	The role of HRM in ICT implementation	S3
	Framework for goals for ICT use in HR	S19
	Framework for ICT effects, enriched with the concept of organizational object and integrating perspective on emergence and enacted practices	S21
Other broad management /business	Structuration theory	S3; S7
	Management strategies	S6
	Game-theoretic model	S6
	Evaluation framework for business process projects	S14
	Knowledge-sharing concept	S15
	Evidence-based health care	S23
	Emancipatory principles and principles of critical social theory	S38
Does not specify	S2, S4, S5, S12, S13, S16, S17, S20, S24, S25, S26, S27, S28, S30, S31, S32, S34, S35, S37, S39, S40, S41	

).

### HRIS types and their functionalities for HRM practices

Most qualifying studies (n = 21) examined dedicated HRIS, comprising one or several modules for supporting particular HRM practices. Sixteen studies focused on generic integrated organizational systems, including modules dedicated to HRM practices. Five did not clarify whether the HRIS were dedicated or components of generic systems (see Table 1).

Descriptions of ICT for managing HR in health organizations lacked a common terminology (see Table 1). Organizational systems that included HRM functions were commonly described as ERP (n = 3), patient classification system (n = 3), or Intranet (n = 2). Dedicated systems were described as HRIS (n = 7), payroll/salary system (n = 4), or electronic-HRM (n = 2). HRIS (n = 3) was used most frequently in studies not specifying whether the system was dedicated or generic.

HRIS support various HRM practices in health organizations. However, as shown in Figure 3, most qualifying studies focus on operational HRM practices (eg, HR administration or scheduling).

### HRIS users

HRIS are designed for a variety of users. The most commonly mentioned user groups were health sector leaders/decision-makers (n = 6), hospital management, HR department/HR professionals, nurses, nurse managers/administrators, and employees (all with n = 5). Less commonly mentioned were health organizations, government//professional authorities, line managers (all with n = 3), staffing clerk/coordinator (n = 2), clinicians, donor agencies, internal temporary employment agencies, rural primary care teams, and nurse educators (all with n = 1). Seven studies did not specify any HRIS user categories.

### Innovation stages

Innovation stage was classified based on our interpretation of a study’s aims and findings rather than any authors’ explicit statements, which often bore little resemblance to the stages described in the study.

Half of the studies (n = 21) focused exclusively on a single innovation stage, mostly on HRIS use (n = 17), with 2 studies focusing on either development or implementation. The other half encompassed several innovation stages, 9 covering development, implementation, and use, 5 development and use, 5 implementation and use, and 2 development and implementation. Table 3

**Table 3.** Innovation stages examined in the included studies

Category		Development	Implementation	Use
Expected benefits		S8, S9, S11, S16, S18, S19, S20, S21, S26, S28, S29, S31, S32, S35, S38, S42		
Factors of influence	Facilitators	S21, S28, S31, S38, S42	S3, S6, S10, S11, S19, S20, S21, S28, S32, S37	S3, S4, S10, S20, S25, S28, S35
	Barriers	S35, S38	S1, S3, S5, S6, S10, S11, S14, S20, S21, S29, S38	S2, S3, S7, S8, S10, S41
	Generic	S8; S10	S5, S8, S11, S19	S21
Approaches to		S21; S30; S38; S42	S6, S8, S11, S14, S21, S29, S32, S37, S38	S2, S5, S8, S18, S20, S21, S22, S23, S24, S25, S26, S27, S31, S32, S34, S37
Recommendations		S21	S7, S9, S21	S9, S21, S25, S27, S30, S32, S34
Outcomes	Realized benefits			S1, S2, S4, S5, S9, S12, S13, S17, S18, S19, S23, S25, S28, S32, S33, S34, S36, S37, S39, S40
	Satisfaction			S5, S8, S9, S15, S17, S27, S28, S32, S35
	Generic			S7, S9, S11, S21
	Downsides			S9

indicates the innovation stages covered and shows that the studies focused mainly on (1) approaches to HRIS use, (2) factors of influence during HRIS implementation, (3) HRIS outcomes, such as realized benefits, and (4) drivers for HRIS.

### Drivers and realized benefits

The majority of studies described HRIS implementation as being driven by expected benefits or goals. The most common related to s*trategic orientation –* being able to use information about HR needs and performance for evidence-based decision-making, to inform HRM policy and planning, or as a means of migrating to a centralized, enterprise-wide HR shared services approach. This was followed by *operational efficiency* – reduction and control of costs, automation or augmentation of manual processes, time saving, and reduced bureaucracy. Improvements in HR *service delivery* were also expected, such as identifying current levels of provision, resolving issues with external service providers, and/or increasing the quality of information in HRIS. Other expectations driving implementation included s*tandardization* of systems, processes, or data; e*mpowerment of managers and/or employees*; *compliance with statutory requirements for data on the health workforce*; and helping to manage macro organizational changes, such as a planned hospital merger. We did not find evidence that health organizations adopted HRIS to improve their *organizational image*, as suggested in Parry and Tyson’s framework.

The most commonly realized benefits of HRIS implementation related to *strategic orientation* and o*perational efficiency improvements,* followed by *empowerment of managers and employees*, improvements in *service delivery*, *standardization, and compliance* with regulatory requirements. Another was improvement in patient care by facilitating minimum standards of nursing care.^43^ One study reported that hospitals using HRIS had lower rates of vascular catheter urinary tract infections.^73^ Generation of interest from other countries^27^ and improved ICT infrastructure^18^ were also reported as beneficial outcomes.

Only 5 studies reported whether projects had achieved their expected benefits, and even fewer described failure of the HRIS to influence specific goals, notably operational efficiency (n = 3), strategic orientation (n = 1), and service delivery (n = 1) (see Table 1 for details).

Only one study (S9) reported specific adverse effects of HRIS implementation within the organization, including negative perceptions of HR roles and increases in supervisors’ workload associated with changing to new HRIS processes. More general adverse effects were mentioned in another study (S21), which described a region-wide HRIS project as a “catastrophic failure”^52^ with multiple negative consequences for contractors and government, including staff strikes and the Minister of Health’s resignation.

### User satisfaction

Three studies reported users being satisfied with the system itself, 1 with its functions, and 4 with the information it provides, although 1 noted dissatisfaction with new HRIS procedures and forms. Two described HRIS satisfaction as being dependent upon ease of use, 2 upon types of users, and 1 each on users’ familiarity with the system, time required to judge systems, whether systems reflect true workload, and time in use, satisfaction increasing with evolving user capabilities and organizational adaptation.

### Factors shaping HRIS development, implementation, and use

Facilitators and barriers were reported across innovation stages (see Table 4

**Table 4.** Summary of influential factors mentioned in the included studies

	Technology	Organization	Project	Environment	Task	Inter- organization	Individual
Facilitators	Development						
			S21, S28, S31, S38	S42			S21, S28
	Implementation						
	S3	S3, S6, S10, S20	S6, S19, S21, S28, S32	S20			S3, S11, S19, S21, S28, S32, S37
	Use						
	S3, S28, S35	S3, S10, S20, S25, S28	S4, S28, S35	S20, S25			S3, S28
Barriers	Development						
		S35, S38			S38	S38	S35
	Implementation						
	S10, S14, S20, S21	S3, S5, S6, S10, S11, S14, S20, S21, S38	S1, S11, S14, S21, S29	S20, S21	S14, S21	S14, S21, S38	S1, S3, S11, S14, S20, S21
	Use						
		S3, S7, S10, S41	S2, S7, S8	S2	S7, S10, S41		S2, S3, S7, S8, S41
Generic	Development						
	S8, S10	S8					S8
	Implementation						
	S19	S11	S5				S5, S8, S11
	Use						
	S21	S21	S21				
Approaches to	Development						
	S30		S38	S21, S42		S30, S38, S42	
	Implementation						
	S6, S11, S21, S32, S38	S29	S6, S8, S11, S14, S21, S29, S38		S37	S8, S14, S21	S8, S32
	Use						
	S2, S5, S8, S18, S24, S25, S26, S27, S32, S34, S37	S26	S21, S31	S26	S20, S22, S23, S26, S27, S31	S31	S5, S31, S32
Recommendations	Development						
	S21	S21	S21	S21	S21		S21
	Implementation						
	S21	S7	S7, S9, S21		S7, S9, S21	S21	S7, S9
	Use						
	S21, S25, S34	S21, S32	S9, S21, S27, S32	S32	S9, S21, S27, S30	S21	S21, S27

). Success was influenced primarily by project-related factors, including governance structure, approaches to project management, and quality of execution, and by individual factors such as stakeholders’ political behaviors and user involvement. Organizational factors, including organizational size, diversity, culture, degree of centralization, and availability of resources, were the most significant barriers. Some studies described technological barriers, including breadth of system functionality, degree of local configuration, and interoperability. Barriers associated with existing HR processes were also mentioned, and several studies recommended simplifying such processes prior to HRIS introduction, although none reported any evidence of this having facilitated a project’s success. Macro-environmental influences, such as political reforms and inter-organizational relationships, were considered very little.

## DISCUSSION

### Summary

The intention of this review was to capture, synthesize, and interpret existing evidence on HRIS in health care organizations. We discovered that research in this area ranges across disciplines and varies widely in terms of its objectives, methods, theoretical orientation, quality, and language. As expected, the evidence base is sparse compared with clinical information systems research. Most studies focus, somewhat uncritically, on the use and realized benefits of HRIS in practice, rather than sociocontextual or technological factors influencing their development, implementation success, or impacts on strategic decision-making or cost-effectiveness. Most research comes from higher-income countries and examines small-scale systems in individual hospital settings. Nevertheless, several higher-quality studies were found, including one national program evaluation, and we were able to adapt and apply existing theoretical frameworks to help organize and interpret the evidence, suggesting that it may be possible to build a more integrated body of research in this area.

### Scope and meaning of HRIS

The plethora of terms used to describe HRIS, and variation across disciplines, suggests a lack of consensus and makes it difficult to build a coherent evidence base. This may explain why a previous systematic review on HRIS in health^64^ did not identify any research prior to 2000, whereas our review, using a broader range of search terms, found 7 such studies. Therefore, we recommend that researchers go beyond obvious keywords (eg, HRIS) when undertaking background research for new projects (for list of relevant keywords, see^9^).

### Types and quality of research

Purely descriptive research was excluded at the screening phase, hence the methodological quality of the included studies was higher than in the literature as a whole.

Most included studies were published in health journals, but many in social science and ICT journals, with some crossing disciplines. Over half were qualitative, and of those reporting quantitative data, none evaluated cost-effectiveness or return on investment. Given the considerable expenditure on HRIS within the heath sector, this gap is surprising, although it reflects a broader evidence deficit in the health informatics literature.^85^^,^^86^

### Use of theory

The use of relevant theories was an important consideration for our assessment of HRIS research. Although many studies mentioned one or more theoretical frameworks, half did not, confirming observations from a previous literature review on HRIS.^87^ Most of the theoretically informed studies were published in social science journals or as academic dissertations. Of the studies mentioning a theoretical perspective, nearly all referred to different ones. As such, in line with clinical systems studies, which seldom build on prior research,^88^ studies on HRIS research in health mostly represent applied projects and do not advance theoretical understanding of HRIS development, implementation, or use.

### International perspectives

The focus of HRIS research has varied across countries in terms of systems, contexts, and priorities. Most studies from high-income countries have focused on small-scale systems in individual hospital settings, with the key users being internal personnel and managers (clinical/nonclinical), although there are notable exceptions, such as a major program evaluation in Australia.^8^ Moreover, nearly all user satisfaction studies have come from high-income countries.

Research from lower-income countries tends to concentrate on open-source HRIS to collect data at the national and regional levels, focusing on health leaders and decision- and policy-makers as the primary system users. Most studies, especially those from low-income countries, prioritize operational aspects of HRM practices, despite WHO recommending in 2001 that effective HR departments should also undertake managerial or strategic HR activities.^89^

We observed a general scarcity of HRIS research in health from East Asia and the Pacific, Eastern Europe, Central Asia, Latin America and the Caribbean, the Middle East and North Africa, South Asia, and sub-Saharan Africa. Moreover, we did not identify any study that compared HRIS projects across countries, supporting the call for more international comparisons of ICT research in health.^90^

### Stages of innovation and evaluation

The majority of existing HRIS studies have concentrated on the use of systems in practice across several innovation stages. Very few focused on the development stage, and even fewer reported measurable outcomes of HRIS projects. While some studies differentiated between expected and realized benefits, we found no rigorous evaluations that compared both systematically. The focus on usage compared to development and impact suggests that the importance of user-centered design for the success of health ICT projects and the need for evaluation have not been fully acknowledged.

### Key messages

HRIS are underrepresented in the health informatics literature, despite their potential to contribute to information-driven learning health systems and the substantial financial investments that are being made in them. Most research is based on softer forms of evidence, and there are important gaps in knowledge about the impacts and cost-effectiveness of these systems, which calls for further research. Interdisciplinarity is a positive characteristic of this literature, in view of the importance of sociotechnical factors for the success of HRIS projects, but the sheer variety of terminologies and theories represents a barrier to building the coherent evidence base needed to translate evidence into practice.

Of the many studies in our review, only 4 looked at the potential for HRIS to support wider aspects of health care and their indirect effects on patient outcomes, despite their having been characterized as “the only class of hospital IS that has a dual beneficial impact [on] patient care [and] operating costs.”^76^

Given the rising cost of health care and the growth in patient traffic, the future sustainability of health systems will depend on making the best use of information to optimize deployment of HR.^3^ Linking the administrative data from HRIS with data on clinical processes and outcomes offers tremendous opportunities to enable real-time and predictive analytics alongside continuous monitoring and evaluation for smart, efficient, and “learning” health systems.^91^

### Limitations

By excluding descriptive HRIS studies, which are published mostly by HR and clinical practitioners, we may have missed applied case studies with valuable insights for the area. The timeline of our review means that some recent studies^92^ are not integrated. While multiple publications have emerged from the United States Agency for International Development’s Capacity and Capacity Plus programs on global health workforce strengthening, we have included 2, the final report for the Capacity project^27^ and the last available report on the Capacity Plus project,^71^ which we believe provide a fair representation of the overall findings of this program and its activities. In common with other systematic reviews, publication bias is a risk, as most of the published studies report only positive results and several were compiled by consulting firms paid by the implementing organization.

## CONCLUSIONS

This review addresses an important gap in the health informatics research literature and can serve as a helpful point of reference for managers planning or implementing HRIS, academics studying health IS, and policymakers or research sponsors considering an investment in health informatics. We also hope that scholars studying HRM practices in health organizations and HRIS in other sectors may find this a useful contribution to the field. We recommend new programs of interdisciplinary research, encompassing economic evaluations, sociotechnical analyses, studies of information flows, and systematic assessments of the impacts of better workforce information on health care efficiency, quality, safety, and patient care, as well as new exploratory research to understand the value of information for driving analytics in support of sustainable and effective health systems.

## Supplementary Material

Supplementary Data
