# Supplementary material for: Human resource information systems in health care: a systematic evidence review
Source: J Am Med Inform Assoc. 2016 Oct 5;24(3):633–54. doi: 10.1093/jamia/ocw141 (PMC5391731; doi:10.1093/jamia/ocw141)
Supplement: Supplementary Data [file ocw141_supp.docx]

#
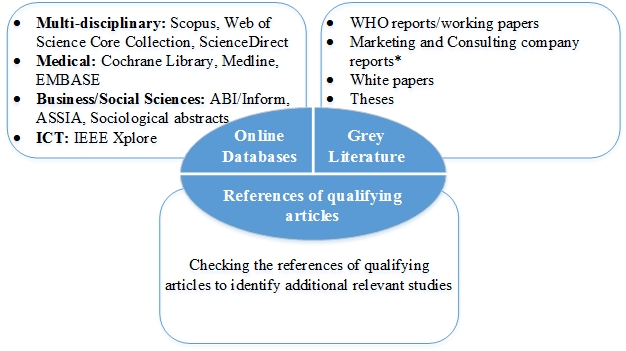
Appendix 1. Search strategy:

*Chartered Institute of Personnel and Development, Deloitte, Ernst & Young, PricewaterhouseCoopers, KPMG, Society for Human Resource Management, Towers Watson, McKinsey & Company, Boston Consulting Group and Sierra-Cedar.

**a) Search query: free field-format**

(Health OR Healthcare OR Hospital* OR Clinic* OR Medic*) AND ("HR management system*" OR "Human resource management system*" OR "Workforce management system*" OR "Personnel management system*" OR "Manpower management system*" OR "Employee management system*" OR "Staff management system*" OR "E-HR" OR "e-HRM" OR eHRM OR HRIS OR “electronic Human resource” OR “HR information system*” OR "HR technolog*" OR "HR management information system*" OR "HR administration system*" OR “Human resource information system*” OR "Human resource management information system*" OR "Human resource administration system*" OR "Workforce information system*" OR "Workforce technolog*" OR "Personnel information system*" OR "Personnel management information system*" OR "Personnel administration system*" OR "Manpower information system*" OR "Manpower management information system*" OR "Employee information system*" OR "Employee management information system*" OR "Staff information system*" OR "Staff management information system*" OR "Staff administration system*" OR “HR information technolog*” OR "HR management technolog*" OR “Human resource information technolog*” OR "Human resource management technolog*" OR "Human resource* technolog*" OR "Personnel Staffing and Scheduling Information Systems" OR "electronic HRM" OR "Virtual HRM" OR "Web-based HRM" OR "HR Portal" OR "HR Online" OR "HR Intranet" OR "E-recruit*" OR "Electronic recruit*" OR "E-employment" OR "Virtual HR" OR "Web-based HR" OR "Business-to-employee" OR "Employee self service")

**b) Search query: Medline format**

1. Health.mp. or Health/
2. Healthcare.mp. or "Delivery of Health Care"/
3. Hospital.mp. or Hospitals/
4. Clinic*.mp.
5. Medic*.mp.
6. 1 or 2 or 3 or 4 or 5
7. "HR management system*".mp.
8. "Personnel Staffing and Scheduling Information Systems"/
9. "Human resource management system*".mp.
10. "Workforce management system*".mp.
11. "Personnel management system*".mp.
12. "Manpower management system*".mp.
13. "Employee management system*".mp.
14. "Staff management system*".mp.
15. "E-HR".mp.
16. eHRM.mp.
17. “e-HRM”.mp
18. HRIS.mp.
19. electronic Human resource.mp.
20. HR information system.mp.
21. HR technology.mp.
22. "HR management information system*".mp.
23. "HR administration system*".mp.
24. Human resource information system.mp.
25. "Human resource management information system".mp.
26. "Human resource administration system*".mp.
27. "Workforce information system*".mp.
28. "Workforce technology".mp.
29. "Personnel information system".mp.
30. "Personnel management information system".mp.
31. "Personnel administration system".mp.
32. "Manpower information system".mp.
33. "Manpower management information system".mp.
34. "Employee information system".mp.
35. "Employee management information system".mp.
36. "Staff information system".mp.
37. "Staff management information system".mp.
38. "Staff administration system".mp.
39. HR information technology.mp.
40. HR management technology.mp.
41. Human resource information technology.mp.
42. Human resource management technology.mp.
43. "Human resource technology".mp.
44. "Electronic HRM".mp.
45. "Virtual HRM".mp.
46. "Web based HRM".mp.
47. "HR Portal".mp.
48. "HR Online".mp.
49. "HR Intranet".mp.
50. E-recruiting.mp.
51. Electronic recruiting.mp.
52. "E-employment".mp.
53. "Virtual HR".mp.
54. "Web-based HR".mp.
55. "Business-to-employee".mp.
56. "Employee self service".mp.
57. 7 or 8 or 9 or 10 or 11 or 12 or 13 or 14 or 15 or 16 or 17 or 18 or 19 or 20 or 21 or 22 or 23 or 24 or 25 or 26 or 27 or 28 or 29 or 30 or 31 or 32 or 33 or 34 or 35 or 36 or 37 or 38 or 39 or 40 or 41 or 42 or 43 or 44 or 45 or 46 or 47 or 48 or 49 or 50 or 51 or 52 or 53 or 54 or 55 or 56
58. 6 and 57

# Appendix 2. Study quality assessment criteria.

| **Category** | **Questions** | **Hints to consider** | **Yes** | **Not Clear** | **No** |
| --- | --- | --- | --- | --- | --- |
| Research objectives | Was there a clear statement of the aims of the research? | Is there a rationale for why the study was undertaken? | S1; S2; S3; S4; S5; S6; S7; S8; S9; S10; S11; S12; S13; S14; S15; S16; S17;  S19; S20; S21; S22; S23; S24; S25; S26; S27; S28; S29; S30; S31; S32; S33; S34; S35; S36; S37; S38; S39; S40; S41; S42 | S18 | N/A |
| Research design | Was the research design appropriate to address the aims of the research? | Has the researcher justified the research design (e.g. have they discussed how they decided which methods to use). | S2; S3; S5; S7; S8; S9; S11; S12; S14; S19; S20; S22; S26; S29; S33; S36; S38 | S1; S4; S6; S15; S16; S21; S23; S24; S25; S27; S28; S30; S31; S34; S35; S40; S41; S42 | S10; S13; S17; S18; S32; S37; S39 |
| Sampling | Was the recruitment strategy appropriate to the aims of the research? | Has the researcher explained how the participants or cases were identified and selected?  Have the researchers explained why the participants or cases they selected were the most appropriate to provide access to the type of knowledge sought by the study?  Was the sample size sufficiently large? | S5; S6; S7; S8; S10; S11; S16; S19; S20; S21; S22; S23; S26; S28; S29; S31; S33; S34; S36; S41 | S2; S3; S4; S9; S12; S13; S14; S15; S17; S18; S24; S25; S27; S30; S35; S38; S40; S42 | S1; S32; S37; S39 |
| Data collection | Was the data collected in a way that addressed the research issue? | Is it clear how data was collected (e.g. semi-structured interviews, focus group etc.)?  Has the researcher justified the methods that were chosen?  Has the researcher made the methods explicit (e.g. is there an indication of how interviews were conducted, did they use an interview guide)?  If the methods were modified during the study, has the researcher explained how and why?  Whether the form of the data is clear (e.g. tape recording, video material, notes etc.) | S3; S4; S5; S6; S7; S8; S9; S16; S19; S22; S23; S24; S26; S28; S29; S30; S31; S38; S40; S41; S42 | S2; S10; S11; S12; S14; S15; S17; S20; S21; S25; S27; S33; S34; S35; S36 | S1; S13; S18; S32; S37; S39 |
| Data analysis | Was the data analysis sufficiently rigorous? | Was there an in-depth description of the analysis process?  If thematic analysis was used, is it clear how the categories/ themes were derived from the data?  Has sufficient data been presented to support the findings?  To what extent has contradictory data been taken into account?  Whether quality control methods were used to verify the results? | S9; S11; S16; S19; S22; S23; S24; S29; S33; S36 | S1; S3; S4; S5; S6; S7; S10; S14; S15; S17; S20; S21; S25; S26; S27; S28; S30; S31; S34; S35; S38; S40; S41; S42 | S2; S8; S12; S13; S18; S32; S37; S39 |
| Reflexivity (research partnership relations/recognition of research bias) | Has the relationship between researcher and participants been adequately considered? | Has the researcher critically examined their own role, potential bias and influence during the formulation of research questions, sample recruitment, data collection, and analysis and selection of data for presentation?  How the researcher responded to events during the study and whether they considered the implications of any changes in the research design? | S2; S38 | S22 | S1; S3; S4; S5; S6; S7; S8; S9; S10; S11; S12; S13; S14; S15; S16; S17; S18; S19; S20; S21; S23; S24; S25; S26; S27; S28; S29; S30; S31; S32; S33; S34; S35; S36; S37; S39; S40; S41; S42 |
| Findings | Is there a clear statement of findings? | Are the findings explicit (e.g. magnitude of effect)?  Has an adequate discussion of the evidence, both for and against the researcher’s arguments, been demonstrated?  Has the researcher discussed the credibility of their findings (e.g. triangulation, respondent validation, more than one analyst)?  Are limitations of the study discussed explicitly?  Are the findings discussed in relation to the original research questions?  Are the conclusions justified by the results? | S1; S2; S3; S4; S5; S6; S7; S8; S9; S10; S11; S14; S16; S17; S19; S20; S21; S22; S23; S24; S25; S26; S27; S28; S29; S30; S31; S32; S33; S34; S35; S36; S38; S39; S40; S41; S42 | S12; S13; S15; S18; S37 |  |
| Value of the research | Is the study of value for research and practice? | Does the researcher discuss the contribution the study makes to existing knowledge or understanding (e.g. do they consider the findings in relation to current practice or relevant research-based literature)?  Does the research identify new areas in which research is necessary?  Does the researcher discuss whether or how the findings can be transferred to other populations, or consider other ways in which the research can be used? | S1; S3; S4; S7; S9; S11; S12; S14; S16; S17; S19; S21; S22; S25; S26; S30; S33; S38; S40; S42 | S2; S5; S6; S8; S10; S13; S15; S18; S20; S23; S24; S27; S28; S29; S31; S32; S34; S35; S36; S37; S41 | S39 |
